# Inspiratory and expiratory sinus arrhythmia in healthy human

**DOI:** 10.14814/phy2.70245

**Published:** 2025-04-02

**Authors:** Pascale Calabrese, Sophie Lambert‐Lacroix

**Affiliations:** ^1^ Université Grenoble Alpes, CNRS, UMR 5525 Grenoble France

**Keywords:** expiration, human, inspiration, respiratory sinus arrhythmia, statistical analyses

## Abstract

Breathing and ECG were recorded in 33 healthy human subjects at rest. The RR interval was labeled according to its occurrence in the respiratory phases: II (both R waves during inspiration), IE (first R wave in inspiration and following R wave in expiration), EE (both R waves during expiration), and EI (first R wave in expiration and following R wave in inspiration). For each subject, II and EE intervals were plotted versus normalized mean inspiratory duration and normalized mean expiratory duration, respectively, and correlation coefficients and slopes of regression lines were calculated. Statistical analyses were conducted to compare these slopes between and within individuals. These relationships appeared to be linear in most cases, although neither the decrease nor the increase in heart rate occurred at the same rate for all subjects. Overall, the slope associated with II intervals was not higher, in terms of absolute values, than the slope associated with EE intervals for the same subject. Our results underscore the difference between inspiratory and expiratory sinus arrhythmia and suggest that the effects of any stimulation presumed to induce changes in vagal cardiac activity should primarily be sought during expiration.

## 1 INTRODUCTION

Respiratory sinus arrhythmia (RSA) is a rhythmical change of the heart rate at the respiratory frequency that is characterized by an acceleration and deceleration of heart rate with inspiration and expiration, respectively. Measurement of RSA may provide an index of vagal cardiac output in humans (Eckberg, 1983). As such, it has been used in numerous psychophysiological, physiological, and clinical studies, as detailed by Berntson et al. (1993). In most investigations, RSA is evaluated over the complete respiratory period without considering inspiration and expiration separately. Indeed, only few studies exist on the changes in RSA in relation with inspiratory and expiratory duration. Strauss‐Blasche et al. (2000) reported that in addition to respiratory rate and tidal volume, RSA can also be modulated by the inspiratory/expiratory time ratio. However, Wang et al. (2013) observed that spectral indexes of heart rate variability (HRV) were not influenced by inspiratory/expiratory time ratio during paced breathing. Friedman et al. (2012) calculated the statistical relationship between the onset of either inspiration or expiration and the timing of R waves before and after these respiratory events in order to evaluate cardio ventilatory coupling. Fonseca et al. (2013) studied the synchrony between heart rate and breathing frequency by using a coherence analysis and they found that “coherence is consistently higher in inspiration than in expiration (p<0.05) for simple and causal coherence from respiration to HRV.”

In each breath, inspiratory and expiratory phases exhibit specific characteristics: within the central nervous system, the activity of the engaged muscles and its resulting mechanics, as well as the difference in duration and intrathoracic pressure (Von Euler, 1986). Furthermore, in a previous study (Calabrese et al., 2000), we partially investigated the effect of baroreceptor stimulation on RSA and concluded that changes in sympathetic and/or vagal outflow during quiet breathing are more likely attributable to respiration itself rather than to the arterial pressure changes associated with respiration. This in itself justifies that RSA during inspiration and expiration be reexamined separately. Our study aims to qualitatively and quantitatively compare changes in the RR interval pattern during inspiration and expiration separately, to gain further insight into the mechanism of RSA. It has been carried out on healthy subjects breathing spontaneously. During each breath, RR intervals were identified based on their position within the respiratory phases. Moreover, the occurrence time of an R wave was calculated in each breath during inspiration (respective expiration) in fraction of normalized inspiratory (respective expiration). This allows comparison of RR interval variations between inspiration and expiration within each subject. Furthermore, the patterns of RR interval variations in each phase were compared across all subjects to determine whether a common pattern can be identified among all subjects.

## 2 MATERIALS AND METHODS

### 2.1 Subjects

In the study 39 healthy volunteers (17 women; mean ± SD height: 1.70 ± 0.1 m; weight: 70.1 ± 12.0 kg) were included. Subjects were recruited among university staff members and were between 19 and 45 years of age (27.7 ± 5.7 years). After a description of the experimental design and protocol, each subject signed an informed consent form. The experimental protocol was examined and approved by the Institutional Ethics Review Board of the University Hospital (CHUGA) in Grenoble, and the study was registered on ClinicalTrials.gov (# NCT02114749). After processing the recordings, six subjects were excluded because their heart rate‐to‐respiratory rate ratio was less than 4. This exclusion is justified by the analytical method described below.

### 2.2 Experimental protocol

In a quiet room and in a comfortable seated position, subjects were asked to relax and to breathe freely with eyes open during the whole recording period. They wore a facemask equipped with a flowmeter (Fleish head No. 1, Emka Technologies, Paris, France) and a differential pressure transducer (163PC01D36, Micro Switch Honeywell, United States) to measure respiratory flow (FLOW). Prior to recordings, leaks from around the mask were checked for with an infrared ${\mathrm{CO}}_{2}$ analyzer (Engström Eliza/Eliza MC, Sweden) and end tidal ${\mathrm{CO}}_{2}$ was subsequently measured continuously using the same apparatus to make sure that subjects have a stable level of breathing. An electrocardiographic signal (ECG, lead II) was obtained covering the whole period of recording. For each subject, a 5‐min recording was performed.

### 2.3 Data acquisition and analyses

**FIGURE 1:**
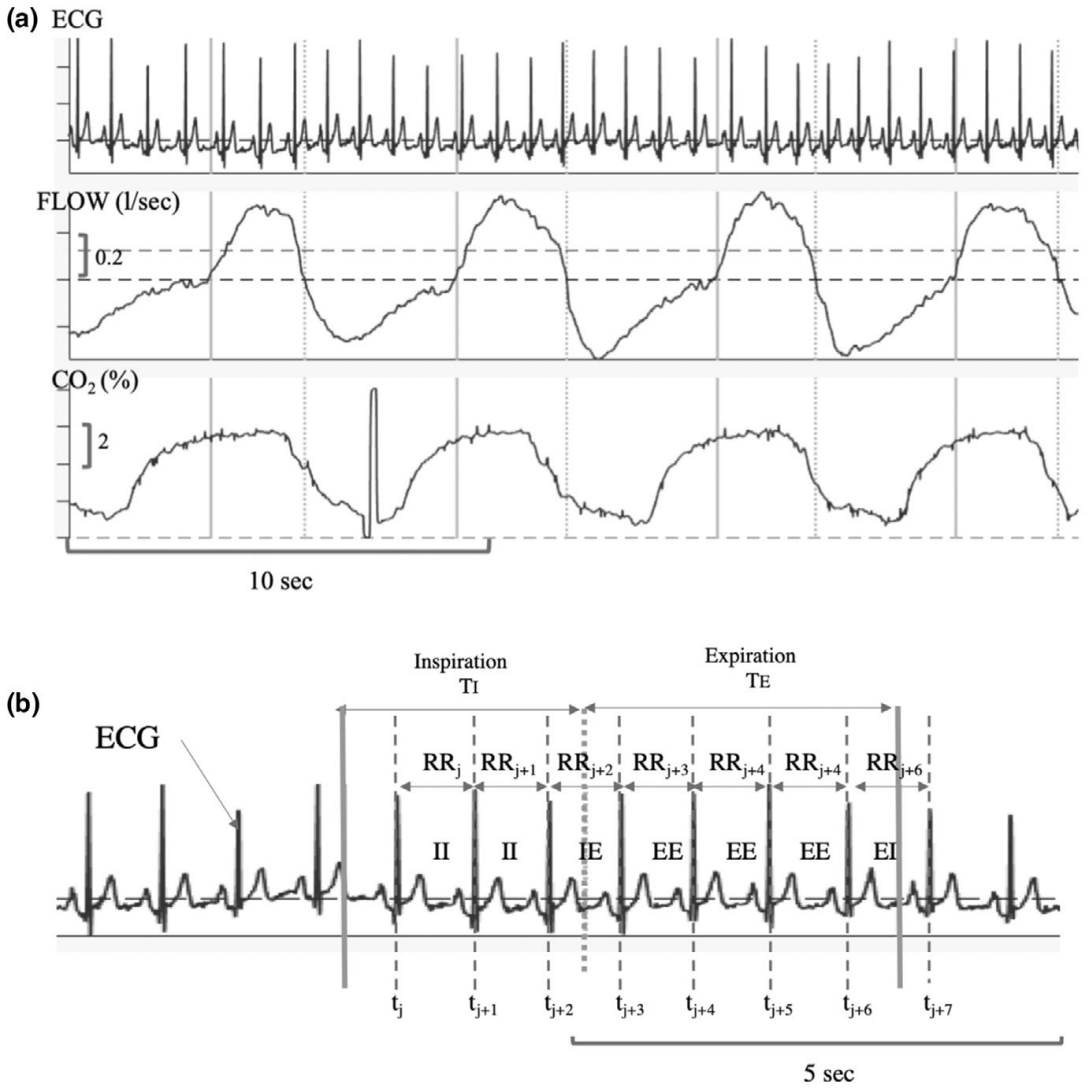
Signals recorded on a healthy subject and labeling of RR intervals. (a) Recorded signals: Electrocardiographic (ECG), airflow (FLOW), and ${\mathrm{CO}}_{2}$ fraction (${\mathrm{CO}}_{2}$). Respiratory phases of each breath were detected: Continuous vertical lines correspond to the beginning and end of respiratory cycle and dotted vertical lines correspond to the transition from inspiratory to expiratory phases. (b) The time when the _*j*_th R waves occurs was identified as $t_{j},$ j=1,…,N, with N: total number of recorded R waves. RR intervals series consisted of values of ${\mathrm{RR}}_{j}$, where ${\mathrm{RR}}_{j}=t_{j+1}-t_{j}$. RR intervals are labeled as follows: II (RR interval is entirely in inspiration), IE (RR interval begins in inspiration and ends in the following expiration), EE (RR interval is entirely in expiration), and EI (RR interval begins in expiration and ends in the following expiration).

Signals recorded on one subject are represented in Figure 1a. FLOW and ECG were synchronously digitized at a rate of 200 Hz with a PowerLab acquisition system and LabChart software (ADInstruments Pty Ltd). As shown in Figure 1a, the beginning and the end of each respiratory cycle were detected (continuous vertical lines) as well as the transition from the inspiratory to the expiratory phases (dotted vertical lines). Inspiratory (Ti) and expiratory (Te) durations, breath period (Ttot), and tidal volume (Vt) were calculated for each breath, and mean values were calculated for each recording.

The ECG signal was processed to extract the RR interval series (Figure 1b). On each recording, the moment when the _*j*_th R waves occurs was identified by $t_{j},$ j=1,…,N, with N: total number of recorded R waves. The RR interval series consisted of the values of ${\mathrm{RR}}_{j}$, where ${\mathrm{RR}}_{j}=t_{j+1}-t_{j}.$ To analyze RR intervals separately during inspiration and expiration, they were categorized based on their position within the respiratory phases, resulting in four categories for each complete breath, as follows:

- II: RR interval is entirely in inspiration (i.e., ${\mathrm{RR}}_{j}$ and ${\mathrm{RR}}_{j+1}$).
- IE: RR interval begins in inspiration and ends during the following expiration (${\mathrm{RR}}_{j+2}$).
- EE: RR interval is entirely in expiration (i.e., ${\mathrm{RR}}_{j+3}$, ${\mathrm{RR}}_{j+4}$, and ${\mathrm{RR}}_{j+5}$).
- EI: RR interval begins in expiration and ends during the following inspiration (${\mathrm{RR}}_{j+6}$).

### 2.4 Breath analysis

In the following figures, the RR intervals were represented according to their labeling: II (▼), IE (Δ), EE (▲), and EI (∇).

**FIGURE 2:**
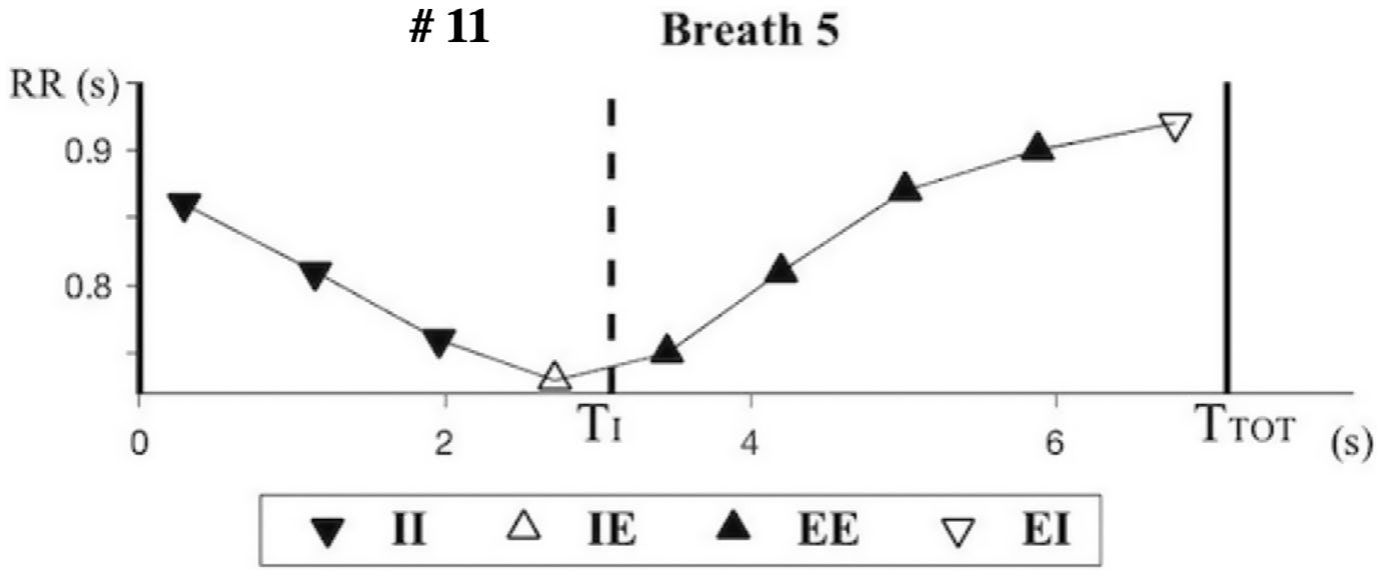
Location of successive RR intervals in one breath.

Figure 2 shows the location of successive RR intervals in one breath: each dot has as abscissa the time of occurrence ($t_{j}$) of an R wave in that breath and as ordinate the RR interval (${\mathrm{RR}}_{j}$) between this R wave and the following R wave.

However, not all breaths exhibit such RR interval distribution. For each recording, the breath percentage was calculated:

1. when the decrease in II intervals was not continuous;
2. when the IE interval is not the shortest during inspiration;
3. when the increase in EE intervals was not continuous;
4. when the EI interval is not the longest during expiration.

### 2.5 Subject analysis

Six recordings were discarded because the heart rate/breathing rate ratio was less than 4, which indicates that four types of RR intervals were not systematically present in the breaths. This analysis was carried out on the remaining 33 recordings.

**FIGURE 3:**
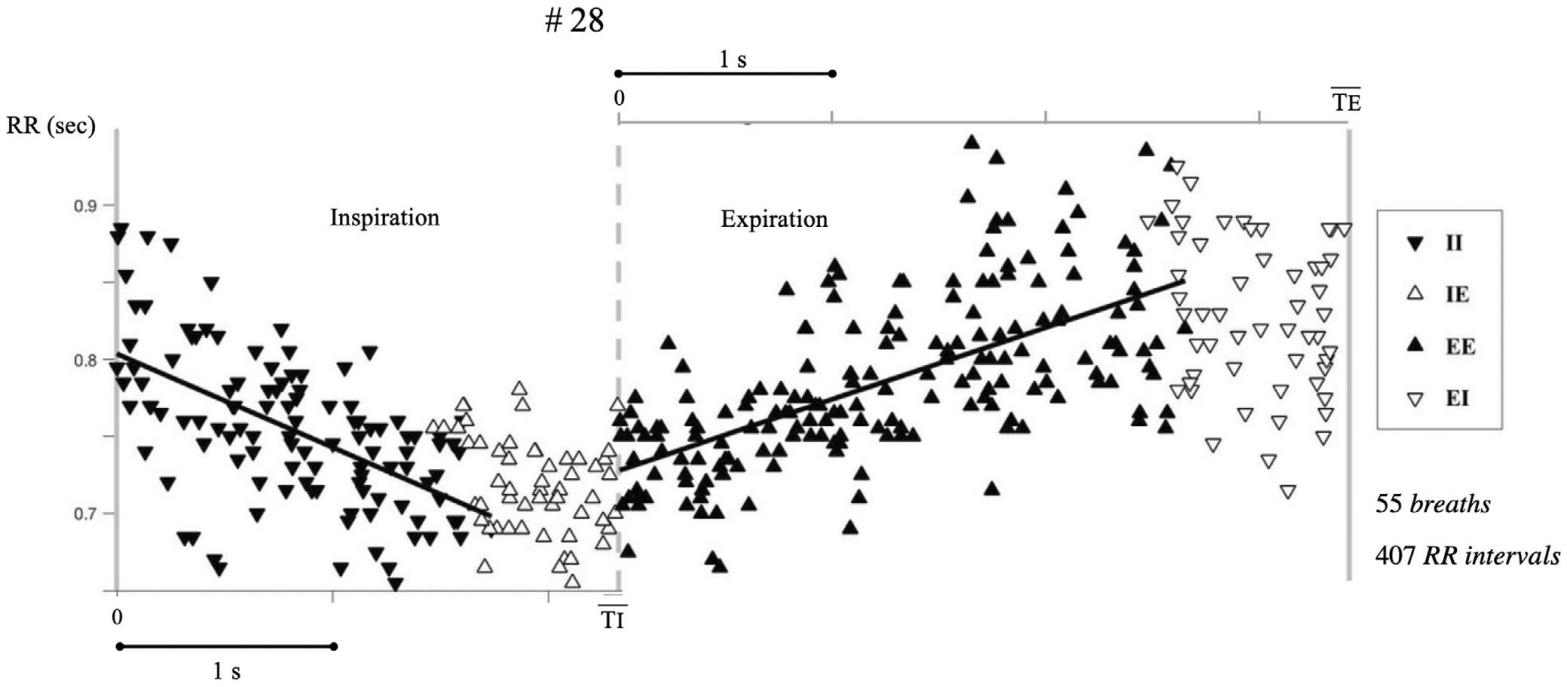
The ordinate of each dot is an RR interval, the abscissa is normalized according to (1) the mean $\bar{TI}$ of the recording when the dot is inspiration and (2) the mean $\bar{TE}$ of the recording when the dot is expiration Linear regression lines were drawn for both II intervals and EE intervals.

Figure 3 shows the position of all RR intervals for Subject 28. In order to illustrate its complete set of data, the time axis was normalized separately for inspiration and expiration. For each point, the ordinate is the RR interval. Normalization in inspiration is obtained by multiplying the occurrence $t_{j}$ time of each R wave by the ratio of the average Ti (labeled $\bar{Tɪ}$) and Ti: $t_{j}\times \bar{Tɪ}/Tɪ.$ Similarly, normalization for $t_{j}$ in expiration is obtained by replacing Ti by Te. Abscissa axes are represented for both inspiratory phase (zero to mean inspiratory time, $\bar{Tɪ}$) and expiratory phase (zero to mean expiratory time, $\bar{Tᴇ}$).

### 2.6 Statistical analyses

Statistical analyses based on models to describe the relationship between RR intervals and the associated normalized times were detailed in the Appendices A, B, C, D. These analyses were conducted to address the following questions:

Question 1 Do II intervals decrease linearly with normalized TI and do EE intervals increase linearly with normalized TE for each subject? If so, are the slopes linearly correlated with ventilatory characteristics?

Question 2 Does the decrease (or increase) in heart rate occur at the same rate for all subjects?

Question 3 Is the slope, in terms of absolute values, associated with II intervals higher than that associated with EE intervals for the same subject?

In order to answer the Question Q1, individual linear regression models was fitted. The p‐values associated with Pearson's correlation coefficients allow us to conclude when to the linear relationship. Concerning the Question Q2, the linear mixed model (see Laird & Ware, 1982) provides a framework to model this type of data and allow to test the presence of random effect on the slope (and intercept). In the absence of random effect on slopes, we can conclude that there is a common slope. In order to answer the Question Q3, we use appropriate tests in multiple linear models that allow modeling both inspiration and expiration intervals.

## 3 RESULTS

Recordings on 33 subjects provided 1961 breaths and 11,077 RR intervals on which the study was performed (Table 1). The number of breaths and of RR intervals by subject is given in Table 1, along with the mean values and standard deviations (SD) of Vt, Ti, Te, and Ttot for each subject.

**TABLE 1:** Cardiorespiratory characteristics and the number (*N*) of breaths and percentage (%) where: (a) EE intervals do not have a continuous increase during expiration, (b) II intervals do not have a continuous decrease during inspiration, (c) IE intervals do not have the lowest RR value in inspiration, and (d) EI intervals don't have the highest RR value in expiration for each subject.

#Subject	Cardiorespiratory characteristics												A		B		C		D	
	Breaths	RR intervals N	RR intervals (s)		Vt (l)		Ti (l)		Te (s)		Ttot (s)		Non‐continuous II intervals		Non‐continuous EE intervals		Not lowest IE intervals		Not highest EI intervals	
	*N*	*N*	Mean	SD	Mean	SD	Mean	SD	Mean	SD	Mean	SD	*N*	%	*N*	%	*N*	%	*N*	%
1	68	274	0.73	0.06	0.39	0.04	1.33	0.15	1.62	0.22	2.95	0.35	1	1.47	2	2.94	16	23.53	35	51.47
2	49	355	0.67	0.05	0.70	0.12	1.98	0.26	2.87	0.39	4.85	0.56	0	0.00	6	12.25	3	6.12	4	8.16
3	49	434	0.68	0.06	0.82	0.09	2.54	0.35	3.55	0.47	6.09	0.69	1	2.00	7	14.29	3	6.12	15	30.61
4	43	222	0.79	0.05	0.48	0.10	1.77	0.19	2.32	0.29	4.08	0.38	1	2.33	1	2.33	3	6.98	7	16.28
5	88	482	0.76	0.05	0.36	0.07	1.51	0.18	2.65	0.70	4.16	0.73	0	0.00	11	12.50	7	7.95	44	50.00
6	55	279	0.89	0.06	0.54	0.09	2.11	0.22	2.43	0.30	4.53	0.43	0	0.00	1	1.82	20	36.36	16	29.09
7	86	411	0.83	0.10	0.43	0.17	1.58	0.25	2.46	0.77	4.04	0.97	0	0.00	3	3.49	0	0.00	25	29.07
8	52	263	0.85	0.10	0.38	0.11	1.74	0.46	2.36	0.57	4.10	0.81	0	0.00	4	7.69	12	23.08	27	51.92
9	37	159	1.11	0.04	0.53	0.08	1.98	0.23	2.78	0.34	4.76	0.51	0	0.00	0	0.00	11	29.73	16	43.24
10	44	225	1.01	0.06	0.42	0.11	2.24	0.44	2.91	0.52	5.15	0.89	0	0.00	2	4.55	7	15.91	21	47.73
11	29	252	0.81	0.05	0.62	0.04	2.85	0.19	4.23	0.45	7.08	0.48	0	0.00	5	17.24	0	0.00	13	44.83
12	87	357	0.83	0.08	0.44	0.02	1.30	0.06	2.13	0.21	3.43	0.24	0	0.00	0	0.00	1	1.15	36	41.38
13	32	295	1.02	0.10	0.90	0.32	3.59	1.03	5.84	1.94	9.43	2.35	4	12.5	24	75.00	12	37.50	28	87.50
14	73	355	0.83	0.03	0.47	0.18	1.78	0.35	2.27	0.35	4.05	0.64	0	0.00	3	4.11	4	5.48	14	19.18
15	38	234	1.12	0.13	0.64	0.15	2.13	0.40	4.60	0.88	6.73	1.24	0	0.00	21	55.26	1	2.63	37	97.37
16	45	397	0.74	0.07	0.74	0.20	2.91	0.72	3.42	0.88	6.33	1.46	2	4.40	23	51.11	14	31.11	16	35.56
17	46	204	0.87	0.08	0.32	0.14	1.35	0.37	2.51	0.75	3.87	1.00	0	0.00	7	15.22	0	0.00	16	34.78
18	78	347	1.07	0.07	0.52	0.08	2.07	0.26	2.69	0.45	4.76	0.68	0	0.00	1	1.28	7	8.97	28	35.90
19	84	398	0.73	0.05	0.35	0.04	1.39	0.17	2.08	0.33	3.47	0.45	0	0.00	3	3.57	0	0.00	6	7.14
20	74	438	0.74	0.05	0.51	0.08	1.94	0.31	2.37	0.38	4.31	0.60	0	0.00	18	24.32	2	2.70	4	5.41
21	76	356	0.91	0.05	0.53	0.05	1.74	0.16	2.52	0.31	4.26	0.40	0	0.00	2	2.63	5	6.58	9	11.84
22	76	369	0.86	0.12	0.38	0.04	1.70	0.25	2.58	0.45	4.29	0.64	0	0.00	5	6.58	2	2.63	20	26.32
23	81	395	0.82	0.04	0.36	0.11	1.58	0.32	2.42	0.75	4.00	0.89	0	0.00	14	17.28	8	9.88	19	23.46
24	65	315	1.03	0.05	0.47	0.12	1.58	0.22	3.43	0.50	5.02	0.65	0	0.00	10	15.38	1	1.54	27	41.54
25	63	360	0.81	0.06	0.38	0.04	1.82	0.22	2.84	0.40	4.66	0.49	0	0.00	4	6.35	17	26.98	18	28.57
26	75	428	0.91	0.11	0.49	0.23	2.35	0.93	2.91	0.79	5.27	1.60	5	6.67	32	42.67	25	33.33	55	73.33
27	58	392	0.79	0.05	0.64	0.35	1.97	0.28	3.33	0.68	5.30	0.94	0	0.00	16	27.59	9	15.52	25	43.10
28	55	407	0.77	0.06	0.96	0.11	2.33	0.23	3.38	0.39	5.71	0.51	0	0.00	7	12.73	2	3.64	24	43.64
29	48	266	0.70	0.05	0.65	0.12	1.65	0.28	2.23	0.36	3.88	0.60	0	0.00	1	2.08	2	4.17	11	22.92
30	41	256	0.99	0.06	0.52	0.08	2.08	0.30	4.08	0.75	6.17	0.91	0	0.00	6	14.63	0	0.00	14	34.15
31	52	261	0.92	0.14	0.58	0.05	1.87	0.19	2.85	0.34	4.72	0.49	0	0.00	8	15.38	1	1.92	38	73.08
32	59	516	0.57	0.05	0.73	0.11	1.85	0.25	3.13	0.36	4.98	0.53	0	0.00	17	28.81	2	3.39	30	50.85
33	51	375	0.78	0.04	0.45	0.05	1.75	0.15	3.96	0.52	5.71	0.55	0	0.00	4	7.84	0	0.00	21	41.18
N:Number of cycles	1957	11,077											14		268		197		719	
Mean	59	*336*	0.85		0.54		1.95		2.96		4.91			4.90		16.42		13.14		38.81
SD	17	86	0.13		0.16		0.49		0.85		1.26			4.19		17.72		12.16		21.44
Max	88	516	1.12		0.96		3.59		5.84		9.43			12.5		75.00		37.50		97.37
Min	29	159	0.57		0.32		1.3		1.62		2.95			1.47		1.28		1.15		5.41
Number of subjects with % ≠ 0														6		31		5		0

### 3.1 Breath analysis

**FIGURE 4:**
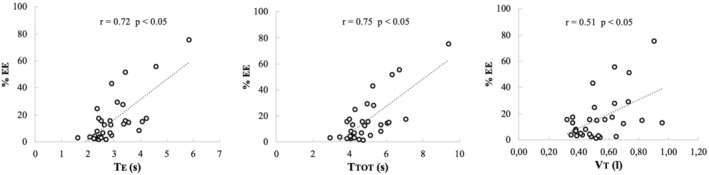
EE % is the percentage of breaths where EE increased continuously during expiration.

The analysis involved calculating for each recording the percentage of breaths that met specific criteria: (1) non‐continuous decrease in II intervals, (2) not the shortest IE interval during inspiration, (3) non‐continuous increase in EE intervals, and (4) not the longest EI interval during expiration (Table 1). In 6 of the 33 subjects, there was at least one breath where the decrease in II intervals was not continuous. The percentage of breaths, calculated for each subject, where II intervals did not decrease continuously during inspiration varied between 1.5% and 12.5%, with a mean value of 4.9% (Table 1). Altogether, in only 13 breaths of the 1957 recorded breaths (0.7%), II intervals did not have a continuous decrease during inspiration. Thus, the continuous decrease of II during inspiration is the main pattern, corresponding to 99.3% of all recorded breaths. In 31 of the 33 subjects, there was at least one breath where the increase in EE interval was not continuous. The percentage of breaths where EE intervals did not have a continuous increase during expiration was calculated for each subject (Table 1). The mean value of this percentage was 16.4%, with extreme values of 1.3% and 75.0%. These percentages were plotted versus Te, Ttot, and Vt (Figure 4). The three calculated coefficients of correlation are significantly different from zero, indicating that the number of breaths with discontinuity in EE interval increase is related to ventilatory characteristics.

Altogether, EE intervals did not have a continuous increase during expiration in 268 breaths out of 1957 recorded breaths (13.7%). Thus, the continuous increase of EE during expiration is a frequent pattern: 86.3% of all recorded breaths. The differences in the sequence of RR intervals according to their position in inspiration or expiration can also be appropriately illustrated on Poincaré plots separately for each phase.

**FIGURE 5:**
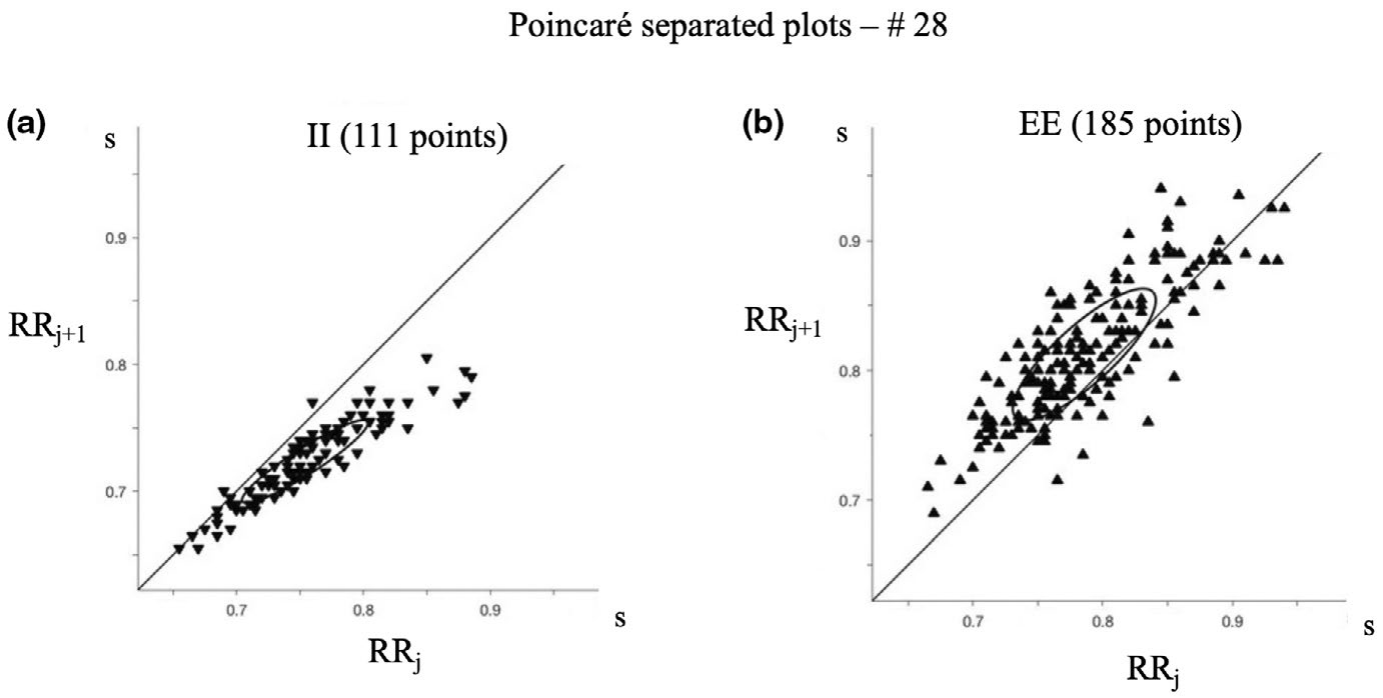
Poincaré plots of EE and II intervals.

In Figure 5a,b, ${\mathrm{RR}}_{j+1}$ versus ${\mathrm{RR}}_{j}$ were plotted in two separated Poincaré plots for II and EE intervals. For II intervals, all points are above the identity line indicating that an interval in inspiration is followed by a shorter interval (Figure 5a), whereas not all EE intervals in expiration are followed by a longer interval (Figure 5b).

It should be noted that there are only one IE and one EI per breath. The IE intervals did not have the lowest RR value in inspiration in all breaths for 28 subjects. The percentage of breaths in these subjects varied between 1.2% and 37.5%, with a mean value of 13.1% (Table 1). Altogether, in only 197 breaths of the 1957 (10.1%), IE intervals did not have the lowest value. Thus, the lowest value of RR intervals is an IE in 89.9% of all breaths. In all subjects, there was at least one breath, where the EI intervals did not have the highest value in expiration. The percentage of breaths, where EI intervals did not have the highest RR value, varied between 5.4% and 97.4% with a mean value of 38.8% (Table 1). Altogether, in 719 breaths of the 1957 (36.7%) EI intervals did not have the highest value. Thus, the highest value of RR intervals is an EI interval in only 63.3% of all breaths.

**FIGURE 6:**
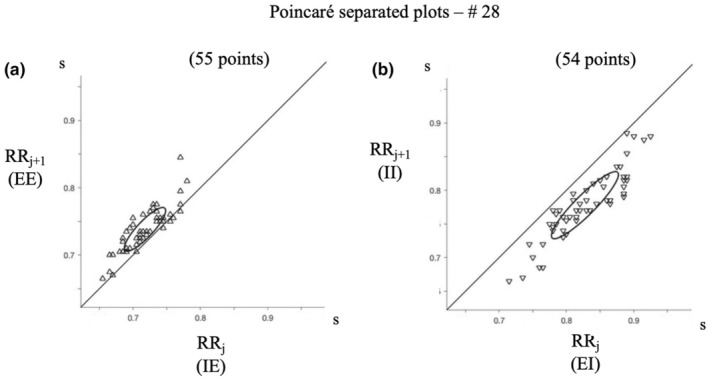
Poincaré plots of EI and IE intervals.

In Figure 6, ${\mathrm{RR}}_{j+1}$ versus ${\mathrm{RR}}_{j}$ were plotted in two separated Poincaré plots for IE (Figure 6a) and EI (Figure 6b) intervals. However, it is noteworthy that in these Poincaré plots, when ${\mathrm{RR}}_{j}$ is an IE interval, ${\mathrm{RR}}_{j+1}$ is an EE interval; similarly, when ${\mathrm{RR}}_{j}$ is an EI interval, ${\mathrm{RR}}_{j+1}$ is an II interval. It can be seen that in Figure 6a, most points are above the identity line, indicating that most IE intervals are followed by a longer EE interval. Similarly in Figure 6b, all points are below the identity line, indicating that all EI intervals are followed by a shorter II interval.

### 3.2 Subject analysis

This analysis based on statistical models which are detailed in Appendices A, B, C, D, is described to address the questions cited in Section 2. Q1: Do II intervals decrease linearly with normalized Ti and do EE intervals increase linearly with normalized Te for each subject? If so, are the slopes linearly correlated with ventilatory characteristics?

**FIGURE 7:**
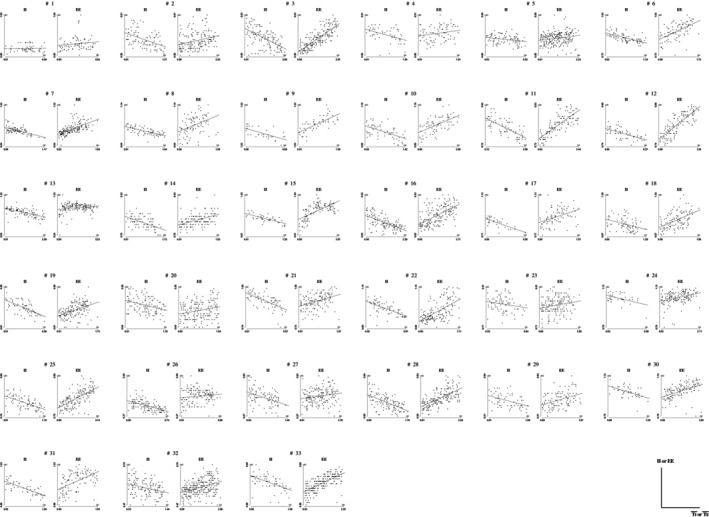
II interval versus $\bar{Tɪ}$ and EE interval versus $\bar{Tᴇ}$ regression lines for all subjects.

In Figure 7, the complete set of II and EE intervals and regression lines, as defined in Figure 3, is plotted for all subjects. We can observe that the relationship between RR intervals and normalized time appears to be linear in most cases, albeit with significant interindividual variability. The slope and the Pearson's correlation coefficient associated with each line were calculated. For II intervals entirely in inspiration, it was found that for 32 of the 33 subjects (exception Subject 1), the coefficient of correlation was negative, and for 31 of the 33 subjects (exception Subjects 1 and 23), it was significantly different from zero at the 0.05 level. For EE intervals entirely in expiration, it was found that in all subjects, the coefficient of correlation was positive, and for 30 of 44 subjects (exception Subjects 1, 4, and 26), it was significantly different from zero at the 0.05 level. On the whole set of subjects, the II regression slopes values did not show a significant correlation with the mean values of Ti, Ttot, Vt, or RR interval for each subject. Similarly, EE regression slopes values did not show a significant correlation with the mean values of Te, Ttot, Vt, or RR interval for each subject.

Q2: Does the decrease (or increase) in heart rate occur at the same rate for all subjects? The likelihood ratio tests in both II and EE conditions for this dataset point toward the presence of a random effect for the intercept and for the slope. Table C.1 (Appendix C) gives the parameter estimates associated with both II and EE intervals. The mean value ± SD of the slope is −0.0771±0.0387 for II interval and 0.0528±0.0369 for EE interval. This result indicates an intersubject dispersion, which allows to conclude that neither the decrease nor the increase in heart rate does occur at the same pace for all subjects. Table C.2 (Appendix C) gives the estimates of the intercept and the slope of each individual fit for both II and EE intervals.

Q3: Is the slope, in terms of absolute values, associated with II intervals higher than the slope associated with EE intervals for the same subject? We observe that Subjects 2, 4, 13, 14, 15, 17, 19, 21, 24, 26, 27, 30, 31, and 33 present slopes (in absolute values) associated with II intervals, which are higher than those associated with EE intervals. For other subjects, the rate of increase and decrease between the two phases is the same. Please note that the linear fits are not significant for Subjects 1, 4, 23, and 26. This means that at least one of the two slopes is considered zero.

## 4 DISCUSSION

Our results show that the pattern of RR interval changes during inspiration and expiration is different not only in terms of acceleration and deceleration but also in terms of progress and control. We are aware that the main caveat in our analysis is the assumption that there exist four different types of RR intervals in each breath. For this reason, we had to discard six subjects (11%), for whom the heart rate/breathing rate ratio was less than 4. Even though, there may be some breaths in which the four different types of RR interval do not exist. Nevertheless, having thoroughly examined our recordings, it appears that this analysis is worth it, as in the great majority of breaths the four types of RR intervals were actually present.

### 4.1 Spontaneous breathing

Our recordings conducted on subjects in spontaneous respiration were intended to analyze the variations in RSA in connection with each naturally occurring respiratory cycle, regardless of its duration and amplitude, along with the Ti/Te ratio. Obviously, this protocol does not allow to fully harness the advantages offered by controlled breathing, where the need to correct breath‐by‐breath variability in respiratory activity is eliminated. However, disparities exist in the assessment of RSA between spontaneous and controlled respiration, and the effects of controlled breathing on RSA magnitude and RSA pattern lack sufficient documentation. The review by Larsen et al. (2010) illustrates differences in RSA amplitude (HF power) ranging from −7% to +89% between spontaneous and controlled respiration, depending on the used paced breathing method. The authors conclude that in comparaison to controlled breathing, slightly more weight should be given to the experiment conducted during spontaneous breathing. Furthermore, all physiological inputs to respiratory control, including those arising from the cardiovascular system are maintained during spontaneous breathing. “While this adds complexity to studying RSA, the complexity is physiological and must be considered.” (Larsen et al., 2010). According to (Ritz, 2009), spontaneous breathing may be favored over paced breathing, because it does not take into account physiological repercussion confusing RSA comprehension and its impact on concurrent physical or mental challenges, thereby restricting research possibilities. However, Hirsch and Bishop (1981) reported that RSA data from spontaneous‐breathing experiments are not significantly different from those of voluntarily controlled breathing experiments.

### 4.2 RR interval in each breath

The originality of our work lies in representing the position of RR intervals in each breath while differentiating both inspiration and expiration in order to separately investigate the evolution of RR intervals in each one of these two phases. We observed that during inspiration, the RR intervals continuously decrease. Figure 5a shows that each II interval is followed by a shorter II interval, and this ongoing decrease is evident in 99.3% for a total number of 1957 breaths. These results suggest that this decrease follows the volume increase and is thus mainly controlled by the inspiratory activity. During expiration, the increase in RR intervals is not always continuous (Figure 5b), and if we consider all the recorded respiratory cycles, even though the increase is continuous in 86.3% of the cycles, there was at least one breath where the increase of the RR interval was not continuous in 31 of the 33 subjects. The correlation between the percentage of such breaths within each recording along with its mean values Vt, Te, and Ttot was found to be statistically significant. This finding underscores that an extended breath duration or an increased tidal volume corresponds to a higher occurrence of breaths exhibiting non‐continuous decreases in EE intervals. Although there is only one IE and one EI interval per respiratory cycle, comparing their values in reference to previous and subsequent intervals reveals the following:

- IE intervals have the lowest values among all intervals in 89.9% of all recorded breaths, and the following EE interval is longer, as illustrated for one subject in the Poincaré figure. This suggests that even though IE spans both inspiration and expiration, the IE interval is still under the influence of inspiration.
- EI intervals have the highest values during expiration in only 63.3% of all breaths. These findings suggest that at the end of expiration, there is a frequent recovery of RR interval shortening. However, the following II interval is always shorter (Poincaré figure). This suggests that at the end of expiration, RR values are not solely controlled by breathing, and as soon as inspiration starts, breathing becomes the leading controller of RR intervals.

Disparities between inspiration and expiration in the interaction between respiration and HRV have been reported by several authors. Fonseca et al. (2013) used coherence analysis on data obtained during spontaneous breathing to emphasize that a single linear model applied to the entire recording overlooks crucial differences between inspiration and expiration. Their results show that simple and causal coherence from respiration to HRV is higher during inspiration than expiration. Another observation that reinforces the relevance of considering inspiration and expiration separately for the investigation of HRV modulation is the evidence that baroreflex responsiveness is different between these two phases. Eckberg and Orshan (1977) observed that by stretching carotid baroreceptors with brief neck suction pulses that vagal responses are greater when baroreceptor stimuli are applied in expiration than in inspiration. Similar results have been reported earlier in animal studies published by Iriuchijima and Kumada (1964) and Haymet and McCloskey (1975). Our hypothesis is that during expiration, changes in heart rate—no longer constrained by the strong inspiratory input—may become “available” for modulation by other factors via the autonomic nervous system. These factors could include stimuli such as stress or environmental changes, which are also likely to influence blood pressure.

### 4.3 Inspiratory and expiratory RR intervals

In most cases, we observed a linear relationship between II interval and normalized Ti, as well as between EE interval and normalized Te. The whole set of II regression slope values did not show any significant correlation with the mean values of Ti, Ttot, Vt, or RR interval for each subject. Similarly, EE regression slope values did not show any significant correlation with the mean values of Te, Ttot, Vt for each subject, suggesting an interindividual variability of inspiratory as well as in expiratory RSA exists, which cannot be explained by respiratory characteristics and cardiac period. Comparison of the slopes between individuals showed that neither the increase of heart rate during inspiration nor the decrease of heart rate during expiration occurs at the same rate for all subjects. Such difference between individuals in RSA has also been reported by Honz Ková et al. (1990): “interindividual variability of circulatory and respiratory spectra was greater than the intraindividual one.” Hirsch and Bishop (1981) observed that the relationships between RSA amplitude and the depth and frequency of breathing characterize the system for each individual when the breathing pattern is voluntarily controlled. Furthermore, Ben Lamine et al. (2004) suggested “that there may exist an individuality of RSA” and Gilad et al. (2005) observed “a possible individuality of RSA pattern characterization, which give rises to a possible ‘finger‐print’ effect.”

A parallel may be drawn with the diversity and individuality of breathing pattern which have been first described by Proctor and Hardy (Proctor & Hardy, 1949)—“the comparison of consecutive cycles or cycles taken on different days from records on any single subject impresses one with the consistency with which an individual pattern is reproduced”—and further developed by Dejours et al. (1961) and Benchetrit (2000). Furthermore, the individuality of breathing has been shown to be preserved over various conditions: over time (Benchetrit et al., 1989), during sleep (Shea et al., 1990), during hypoxia and mild exercise (Eisele et al., 1992), during ventilatory resistive loading (Calabrese et al., 1998), and during volitional hyperventilation (Besleaga et al., 2016).

### 4.4 Slope of II intervals versus slope of EE intervals for the same subject

Following the observation of interindividual differences in II and EE interval slopes, we compared the absolute values of these slopes for each subject. It was found that II interval slopes are higher than EE ones only in 14 of the 33 subjects. Thus, we cannot conclude that the rate of RR interval decrease during inspiration is higher than the rate of RR interval increase during expiration. These results may appear contradictory with respect to heart rate asymmetry reported by several authors, Guzik et al. (2006), Piskorski and Guzik (2011), Bauer et al. (2006), Porta et al. (2008). It must be underlined that these studies were performed on continuous series of RR intervals regardless of breathing while our analysis is performed discontinuously focusing on a breath‐by‐breath basis separated into inspiratory and expiratory phases. Although we did not observe a difference between the absolute values of slopes during inspiration and expiration, it is worthwhile to consider differences between inspiration and expiration, as reported above: during inspiration, the RR intervals continuously decrease, whereas during expiration, the increase in RR intervals is not always continuous, especially when breath duration is long. Such pattern of variation in RR interval during expiration may be explained not only by the fact that, as reported by Eckberg (1983), “a respiratory modulation of the quantity, periodicity, and phase of cardiac vagal efferent activity [exists] and that the vagal firing occurs during expiration” but also by the Eckberg (2003) “wide range of evidence which suggests that respiratory activity gates the timing of autonomic motoneurone firing, but does not influence its tonic level.”

## 5 CONCLUSION

Our observations show the differences between inspiratory and expiratory sinus arrhythmia and underscore the importance of considering respiratory phases when using RSA as an index of cardiac vagal activity. Specifically, the effects of any stimulation presumed to induce changes in vagal cardiac activity should primarily be sought during expiration. Since our study presents findings from healthy subjects, it could serve as a reference for comparing the changes in inspiratory and expiratory RSA observed in cardiac and respiratory pathological conditions.

## AUTHOR CONTRIBUTIONS

Pascale Calabrese conceived and designed research, performed experiments, and drafted the manuscript. Sophie Lambert‐Lacroix performed statistical analysis and drafted statistical results. Pascale Calabrese and Sophie Lambert‐Lacroix analyzed data and prepared figures. Pascale Calabrese and Sophie Lambert‐Lacroix edited and revised the manuscript. Pascale Calabrese and Sophie Lambert‐Lacroix approved the final version of the manuscript.

## FUNDING INFORMATION

None.

## CONFLICT OF INTEREST

No conflicts of interest, financial or otherwise, are declared by the authors.

## Figures and Tables

**FIGURE C1:**
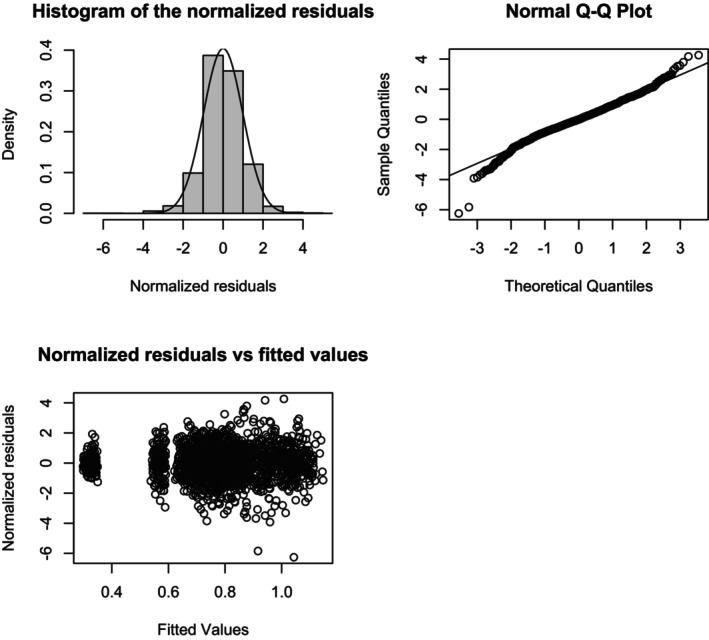
Residuals analysis: Diagnostic plots for the II interval model.

**FIGURE C2:**
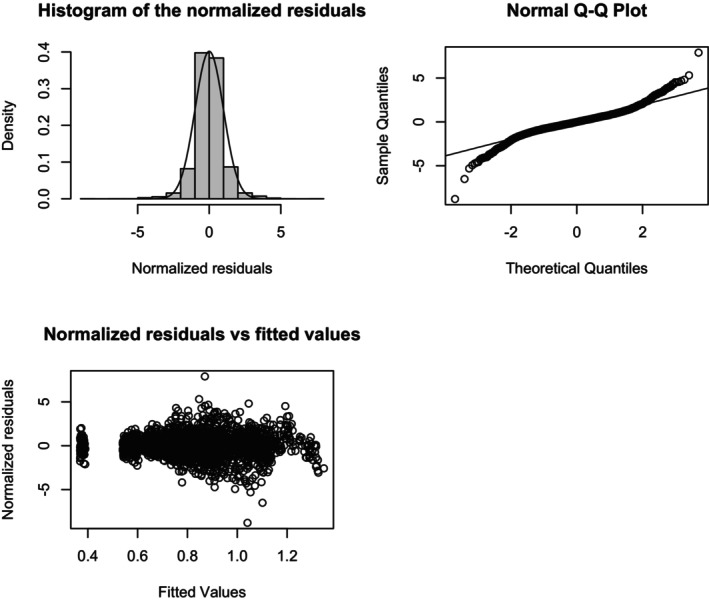
Residuals analysis: Diagnostic plots for the EE interval model.

**FIGURE C3:**
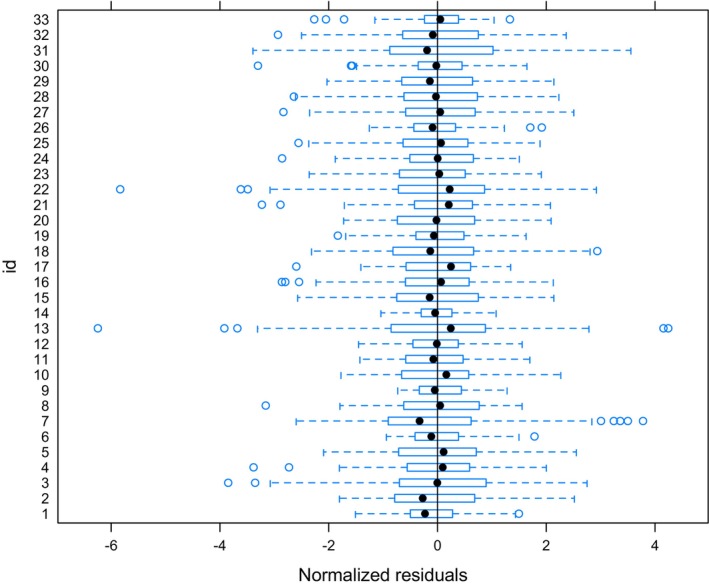
Residuals analysis: Individual boxplots of the normalized residuals per subject for II interval.

**FIGURE C4:**
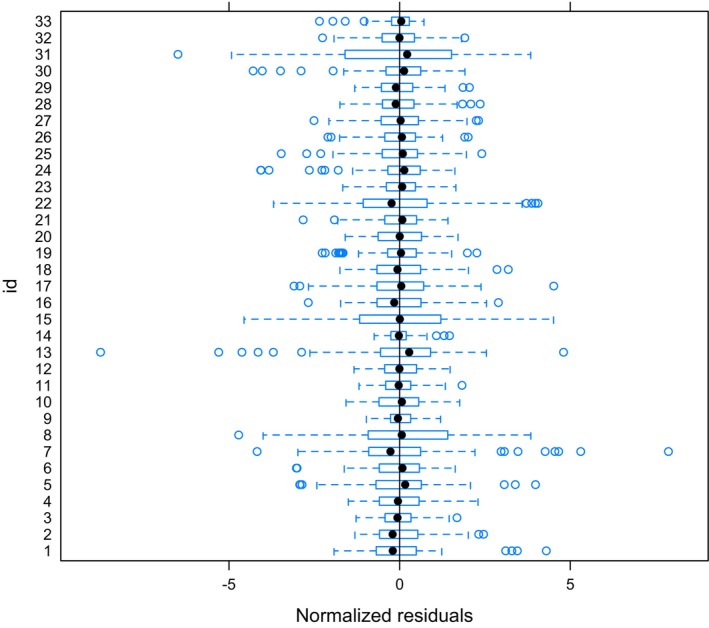
Residuals analysis: Individual boxplots of the normalized residuals per subject for EE interval.

## APPENDIX A

### A.1 Notations

In the sequel, we need to introduce the following notations:

- i: subject index (i=1,…,33).
- ℓ: respiratory interval index, ℓ=II, if the associated RR interval is entirely in inspiration, and ℓ=EE if the associated RR interval is entirely in expiration.
- $n_{i\ell }$: number of RR intervals for subject i and in ℓ interval.
- k: index of RR intervals ($k=1,\ldots ,n_{i\ell }$).
- ${\mathrm{RR}}_{\mathrm{ik}}^{\ell }$: _*k*_th RR intervals for subject i in ℓ intervals.
- $t_{\mathrm{ik}}^{\ell }$: normalized time associated with the ${\mathrm{RR}}_{\mathrm{ik}}^{\ell }$ interval.

## APPENDIX B

### B.1 Answer to the Question Q1: Individual linear fits

Let us recall that Figure 7 represents the dots $\left(t_{\mathrm{ik}}^{\ell },{\mathrm{RR}}_{\mathrm{ik}}^{\ell }\right),$ $k=1,\ldots ,n_{i\ell },$ for each subject i and respiratory interval index ℓ. Linear regression models was fitted to each dots cloud. We can observe that the relationship between normalized times and RR intervals seems linear in most cases but with strong interindividual variability. On the other hand, the linear fits are not significant for Subject 1 for II and EE intervals, for Subjects 4 and 26 for EE intervals, and for Subject 23 for II intervals.

Given that the linear relationship seems to justify for most cases, it may be relevant to study the dependence between the slopes of the different linear fits and the ventilatory characteristics. For example, do the slopes (in absolute values) decrease when the amplitude of the cycle (mean of Vt) increases? When inspiratory (mean of Ti) or expiratory (mean of Te) durations increases? … Table B1 gives the Pearson's correlation coefficients r between slopes associated with II and EE intervals and ventilatory characteristics with associated p‐values. It can be observed that no linear relationship is significant. The plots (not shown here) indicate that no other types of relationship exist.

**TABLE B1:** Pearson's correlation coefficients r between slopes and cardioventilatory characteristics with p‐values.

	$\bar{Tɪ}$		$\bar{T\mathrm{ᴛᴏᴛ}}$		$\bar{Vᴛ}$		$\bar{\mathrm{RR}}$	
	r	p‐value	r	p‐value	r	p‐value	r	p‐value
II slopes	0.258	0.147	0.139	0.440	0.262	0.140	−0.294	0.097

	$\bar{Tᴇ}$		$\bar{T\mathrm{ᴛᴏᴛ}}$		$\bar{Vᴛ}$		$\bar{\mathrm{RR}}$	
	r	p‐value	r	p‐value	r	p‐value	r	p‐value
EE slopes	−0.247	0.166	−0.263	0.139	−0.247	0.165	0.203	0.258

## APPENDIX C

### C.1 Answer to the Question Q2: Linear mixed‐effects models

The linear mixed model (see Laird & Ware, 1982) provides a framework to model this type of data and to respond to the Question Q2. Indeed, this model introduces centered random effects on intercept and slope and allow to model the variability of the response among the different subjects. By adjusting this type of model, we can test the presence of random effects on the slope (and intercept). In the absence of random effects on slopes, we can conclude that there is a common slope. Otherwise, such models allow to have access to the average intercept and the average slope as well as the intraindividual variability.

#### C.1.1 Models' description

In this approach, we consider two different models fitted separately according to inspiration and expiration intervals:

$$\begin{array}{l} \;{\mathrm{RR}}_{\mathrm{ik}}^{\mathrm{II}}=\beta_{0}^{\mathrm{II}}+B_{0,i}^{\mathrm{II}}+\left(\beta_{1}\mathrm{II}+B_{1,i}^{\mathrm{II}}\right)x_{\mathrm{ik}}^{\mathrm{II}}+\epsilon_{\mathrm{ik}}^{\mathrm{II}},\;i=1,\ldots ,33,\;,k=1,\ldots ,n_{\mathrm{iII}},\\ {\mathrm{RR}}_{\mathrm{ik}}^{\mathrm{EE}}=\beta_{0}^{\mathrm{EE}}+B_{0,i}^{\mathrm{EE}}+\left(\beta_{1}^{\mathrm{EE}}+B_{1,i}^{\mathrm{EE}}\right)x_{\mathrm{ik}}^{\mathrm{EE}}+\epsilon_{\mathrm{ik}}^{\mathrm{EE}},\;i=1,\ldots ,33,\;,k=1,\ldots ,n_{\mathrm{iEE}}, \end{array}$$

where $\beta_{0}^{\ell }$ and $\beta_{1}^{\ell }$ are fixed effects (intercept and slope) and $B_{0,i}^{\ell }$ and $B_{1,i}^{\ell }$ are random effects (intercept and slope). The random effects are supposed to be random variables resulting from centered Gaussian law of variance $\sigma_{j,\ell }^{2},$ j=0,1. Thus the slopes are modeled by Gaussian random variables of mean $\beta_{1}^{\ell }$ and variance $\sigma_{1,\ell }^{2},$ ℓ=II, EE. The random variables $B_{0,i}^{\ell }$ and $B_{1,i}^{\ell }$ are assumed to be independent between subjects but not between themselves. There may be a correlation between $B_{0,i}^{\ell }$ and $B_{1,i}^{\ell }$, that we will note $\rho_{\ell }.$ The residuals $\epsilon_{\mathrm{ik}}^{\ell }$ are independent random variable (of $B_{0,i}^{\ell }$ and $B_{1,i}^{\ell }$ and between themselves) with Gaussian law of mean 0 and variance $\sigma_{\ell }^{2}.$

In both these models, we can test if the slope and/or the intercept are random. To confirm the validation of these models, we use the classical plots for diagnostic purposes: normalized residuals histogram, normal QQ‐plot, normalized residuals versus fitted values plot, and normalized residuals boxplot per subject. For more details on the theoretical aspects as well as the practical aspects, the reader is referred to (Pinheiro & Bates, 2000).

#### C.1.2 Results

We use the lme function of the nlme R‐package to fit models. We use the maximum likelihood estimation criterion by specifying method = “ML”. This procedure makes it possible to obtain model parameters estimates (${\hat{\beta }}_{0}^{\ell }$, ${\hat{\beta }}_{1}^{\ell }$, ${\hat{\sigma }}_{\ell }$, ${\hat{\sigma }}_{0,\ell }$, ${\hat{\sigma }}_{1,\ell }$, and ${\hat{\rho }}_{\ell }$) as well as the different intercepts and slopes associated with each subject. These estimated coefficients are obtained by adding together the fixed effects estimates and the corresponding random‐effects estimated by the best linear unbiased predictions (BLUPs). To compare several nested models (for instance to test if the slope and/or the intercept are random or not), we use the anova function which performs likelihood ratio tests.

Residuals analysis of both models is illustrated in Figures C1, C2, C3, C4. The histogram of the residuals and the normal QQ‐plot suggest that the residuals fit the normal distribution reasonably well, except for the extreme tails. The residuals versus fitted values plot does not highlight any residual structure. It can be observed that the residuals are less variable for II interval than for EE interval. Concerning the individual residual analysis, the behavior is quite similar for II interval. On the other hand, for the EE interval, it can be noted that Subjects 8, 15, 22, and 31 have more scattered residuals than for the other subjects.

The likelihood ratio tests indicates for both II and EE intervals, the presence of a random effect for the intercept and for the slope (p‐value less than 0.001 in all 4 cases), that is $\sigma_{j,\ell }^{2}\neq 0,$ j=0,1 and ℓ=II, EE. In particular, this allows us to conclude that the decrease or increase in heart rate does not occur at the same rate for all subjects. Table C1 gives the parameters estimations associated with both intervals. The value ${\hat{\sigma }}_{1,\mathrm{II}}=0.0387$ (resp. ${\hat{\sigma }}_{1,\mathrm{EE}}=0.0369$) gives the dispersion of the various slopes around ${\hat{\beta }}_{1}^{\mathrm{II}}=-0.0771$ for II interval (resp. ${\hat{\beta }}_{1}^{\mathrm{EE}}=0.0528$ for EE interval); what measures the intersubject dispersion. Table C2 gives the estimations of the intercept and the slope of each individual fit for both II and EE intervals.

**TABLE C1:** Estimation of the parameters associated with both linear mixed models for II and EE intervals.

Interval	${\hat{\beta }}_{0}^{\ell }$	${\hat{\beta }}_{1}^{\ell }$	${\hat{\sigma }}_{\ell }$	${\hat{\sigma }}_{0,\ell }$	${\hat{\sigma }}_{1,\ell }$	${\hat{\rho }}_{\ell }$
ℓ=II	0.8610	−0.0771	0.0405	0.1577	0.0387	−0.3270
ℓ=EE	0.7981	0.0528	0.0571	0.1527	0.0369	0.1330

**TABLE C2:** Intercept and slope estimations for each subject associated with both linear mixed models.

Subject	II interval		EE interval	
	Intercept	Slope	Intercept	Slope
1	0.7222052	−0.03391743	0.7345605	0.03830651
2	0.7087796	−0.05817149	0.6413913	0.02132613
3	0.7437017	−0.05921636	0.6071219	0.05678199
4	0.8214142	−0.06568651	0.7785665	0.02402421
5	0.7757049	−0.05309469	0.7443669	0.02162914
6	0.8969967	−0.05634512	0.8534817	0.07471713
7	0.8768035	−0.14483677	0.7621661	0.13663074
8	0.8663244	−0.09878521	0.8175521	0.09470333
9	1.1122512	−0.06489283	1.0881863	0.05451542
10	1.0280380	−0.06095123	0.9834480	0.05451698
11	0.8658106	−0.05135200	0.7577970	0.04159401
12	0.8330448	−0.09506343	0.7899666	0.13230618
13	1.0532199	−0.05583353	1.0388082	0.01288602
14	0.8514428	−0.04884457	0.8165278	0.02809406
15	1.1471327	−0.11648963	1.0689639	0.07573428
16	0.7685998	−0.05044700	0.7068456	0.04642216
17	0.8768995	−0.12253198	0.8459195	0.06883527
18	1.0998055	−0.06597706	1.0363230	0.06935208
19	0.7925871	−0.11498255	0.6915319	0.05238929
20	0.7803504	−0.03914608	0.7086710	0.02335643
21	0.9571992	−0.10275532	0.8756125	0.04056164
22	0.9563789	−0.16618339	0.7447448	0.13814296
23	0.8415559	−0.04308570	0.8034046	0.01915368
24	1.0560614	−0.09330550	1.0050211	0.02773560
25	0.8383343	−0.07900682	0.7611628	0.06401670
26	0.3507995	−0.07132366	0.3701750	0.02317675
27	0.8248933	−0.05908672	0.7808725	0.01431585
28	0.8050546	−0.06221460	0.7274630	0.04766647
29	0.7272372	−0.06067475	0.6748576	0.03831394
30	1.0409110	−0.08111935	0.9449327	0.03189472
31	0.9896161	−0.17435461	0.8887796	0.12060059
32	0.5922689	−0.03738771	0.5410841	0.02365732
33	0.8130499	−0.05865217	0.7463687	0.02393792

## APPENDIX D

### D.1 Answer to the Question Q3: Rate comparison tests

#### D.1.1 Tests' description

Question Q3 concerns the following question: is the slope associated with II interval higher (in absolute values) than the slope associated with EE interval for the same subject? Since we have observed in the previous section that we could not put slopes common to all individuals, we have to have an individual approach to answer this question.

For each subject i, we consider the model

$$RR_{\mathrm{ik}}^{\ell }=\beta_{0,i}^{\mathrm{EE}}1_{\left\{\ell =\mathrm{EE}\right\}}+\beta_{0,i}^{\mathrm{II}}1_{\left\{\ell =\mathrm{II}\right\}}+\left(\beta_{1,i}^{\mathrm{EE}}1_{\left\{\ell =\mathrm{EE}\right\}}+\beta_{1,i}^{\mathrm{II}}1_{\left\{\ell =\mathrm{II}\right\}}\right)t_{\mathrm{ik}}^{\ell }+\epsilon_{\mathrm{ik}}^{\ell },$$

For ℓ=II,EE, $k=1,\ldots ,n_{i\ell }.$ The variables $\epsilon_{\mathrm{ik}}^{\ell }$ are supposed to be independent random variable with Gaussian law of mean 0 and variance $\sigma_{i\ell }^{2}.$ Note that we do not assume that the variances $\sigma_{\mathrm{iII}}^{2}$ and $\sigma_{\mathrm{iEE}}^{2}$ are the same because there is no a priori reason for them to be. In order to answer the previous question we must carry out the test $H_{0}:\beta_{1,i}^{\mathrm{EE}}=-\beta_{1,i}^{\mathrm{II}}$ against $H_{1}:\beta_{1,i}^{\mathrm{EE}}<-\beta_{1,i}^{\mathrm{II}},$ for each subject i. If we reject the null hypothesis the answer to the question will be yes. This test is carried out in two steps; first, we must test the equality of the variances $\sigma_{\mathrm{iII}}^{2}$ and $\sigma_{\mathrm{iEE}}^{2}$ with Goldfeld‐Quandt test. Secondly, if the variances are considered equal, we perform a Student test otherwise an asymptotic Normal test.

#### D.1.2 Results

Table D1 gives p‐value associated with the test to compare the slopes. In particular, we give “p‐value” associated with the Goldfeld–Quandt test and the “p‐value” associated with the Student test or asymptotic Normal test according to the result on the equality of residual variances (G–Q p‐value greater or less than 0.05). If the p‐value associated with the Student test or asymptotic Normal test is greater than 0.05 then the slopes are the same. We observe that the Subjects 2, 4, 13, 14, 15, 17, 19, 21, 24, 26, 27, 30, 31 and 33 present slopes associated with II interval higher (in absolute values) than the slopes associated with EE interval. For other subjects, the rate of increase and decrease between the two intervals can be considered as the same.

**TABLE D1:** p‐values associated with tests to compare the slopes between II and EE intervals. The row “G–Q p‐value” corresponds to the Goldfeld‐Quandt test and the row “ST p‐value” corresponds to the Student test or asymptotic Normal test according to the result on the equality of residual variances (G–Q p‐value greater or less than 0.05). The ST p‐value lower than 0.05 are in bold corresponding to subject with slopes associated with II interval higher (in absolute values) than the slopes associated with EE interval.

Subject	1	2	3	4	5	6	7	8	9	10
G–Q p‐value	0.00	0.51	0.01	0.65	0.02	0.00	0.00	0.00	0.46	0.57
ST p‐value	0.83	**0.00**	0.42	**0.03**	0.09	0.95	0.27	0.46	0.41	0.36

Subject	11	12	13	14	15	16	17	18	19	20
G–Q p‐value	0.83	0.03	0.07	0.45	0.00	0.02	0.00	0.42	0.10	0.29
ST p‐value	0.11	0.92	**0.00**	**0.02**	**0.01**	0.33	**0.01**	0.62	**0.00**	0.11

Subject	21	22	23	24	25	26	27	28	29	30
G–Q p‐value	0.46	0.01	0.53	0.05	0.09	0.00	0.64	0.75	0.46	0.07
ST p‐value	**0.00**	0.09	0.23	**0.01**	0.19	**0.00**	**0.00**	0.07	0.14	**0.04**

Subject	31	32	33							
G–Q p‐value	0.00	0.61	0.79							
ST p‐value	**0.02**	0.11	**0.00**							

Let us recall that he linear fits are not significant for Subjects 1, 4, 23, and 26. This means that at least one of the two slopes (II or EE) is considered zero. Specifically for subject 1, both slopes (II and EE) are considered zero. For subjects 4 and 26, only the slope associated with EE is considered zero, while for subject 23, only the slope associated with II is considered zero. This aligns with the results obtained from the slope comparison test, which shows that subjects 4 and 26 have slopes associated with the II interval that are higher in absolute value than those associated with the EE interval.

Note that the test statistic is based on the deviation between the two estimated slopes but is normalized by a quantity related to the variability of the data around the model. Thus, one could conclude for a certain subject that there is a difference for a smaller gap compared to another subject. For instance, the gap between the two estimated slopes is equal to 0.018 for Subject 14 while it is equal to 0.027 for Subject 5 for which we conclude that there are no difference. The standard deviation common to II and EE for Subject 14 is estimated at 0.02 while for Subject 5 the estimate of the standard deviation is of the order of 0.04 for II and 0.05 for EE.

Contrary to what is expected, the estimated slope associated with II interval is lower in absolute value than that associated with EE interval for Subjects 1, 6, 12, and 18. Note that for these four individuals, a bilateral test at a risk of 5% leads to the same conclusion as for the unilateral test at a risk of 5%, namely that for these four subjects, the slopes can be considered equal.
